# Impact evaluation of the Urban Health Initiative in urban Uttar Pradesh, India

**DOI:** 10.1016/j.contraception.2016.02.031

**Published:** 2016-06

**Authors:** Pranita Achyut, Aimee Benson, Lisa M. Calhoun, Meghan Corroon, David K. Guilkey, Essete Kebede, Peter M. Lance, Anurag Mishra, Priya Nanda, Rick O'Hara, Ranajit Sengupta, Ilene S. Speizer, John F. Stewart, Jennifer Winston

**Affiliations:** a International Center for Research on Women, Asia Regional Office; b Carolina Population Center at the University of North Carolina at Chapel Hill

**Keywords:** Family planning, Impact evaluation, Urban, India, Longitudinal

## Abstract

**Objectives:**

The Urban Health Initiative (UHI) was initiated in 2009 with the goal of increasing family planning (FP) use among the poor in urban areas of Uttar Pradesh, India. The Measurement, Learning & Evaluation project (MLE) was tasked with rigorous impact evaluation of the UHI. This paper presents the impact evaluation findings of the UHI program.

**Study design:**

The MLE design includes a longitudinal sample of women and health facilities with baseline (2010) and endline (2014) data collection in six cities in Uttar Pradesh, India. At baseline, samples representative of women in each city were selected with oversampling of the poor. Eighty-four percent of women interviewed at baseline were reinterviewed 4 years later at endline. The longitudinal data support a within/fixed-effects approach to identification of program impact on changes in modern FP use.

**Results:**

Impact evaluation results show significant effects of exposure to both demand and supply side program activities. In particular, women exposed to brochures (marginal effect: 6.96, p < .001), billboards/posters/wall hangings (marginal effect: 2.09, p < .05), and FP on the television (marginal effect: 2.46, p < .001) were significantly more likely to be using a modern method at endline. In addition, we found borderline significance for being exposed to a community health worker (marginal effect: 1.66, p < .10) and living close to an improved public and private supply environment where UHI undertook activities (marginal effects and p values: 2.48, p < .05 and 1.56, p < .10, respectively).

**Conclusions:**

UHI program activities were designed to complement the Government of India's strategies aimed at ensuring access to and provision of FP to urban poor populations. The effective demand- and supply-side strategies of the UHI program are therefore likely to be sustainable and scalable to other urban areas in India.

**Implications statement:**

Findings from this study are important for designing sustainable and scalable FP strategies for urban India where increases in FP use will be relevant for meeting international FP targets.

## 1 Introduction

The 2012 London Summit on Family Planning encouraged world leaders to make financial and political commitments to improve access to and use of family planning (FP). The Summit sparked a global FP agenda to achieve 120 million new users of modern contraception by 2020 (termed “120 by 20”) [1]. Following the Summit, the Government of India (GoI) pledged to commit over US$2 billion to provide FP services to 48 million new users. This constitutes 40% of the overall FP2020 target [2]. This increase in new users would raise the India national modern contraceptive prevalence rate (mCPR) from an estimated 48.1% in 2012 to 63.7% in 2020 [2].

Nearly twelve and a half million of the new Indian users are expected to come from the state of Uttar Pradesh (UP) [2]. With a population of nearly 200 million, UP is the most populous state in India and has some of the lowest levels of modern contraceptive use. Historically, there have been extensive efforts in UP to reduce population growth and increase FP use. For over two decades (1992–2012), the Innovations in Family Planning Services (IFPS) project, supported by GoI and the United States Agency for International Development, undertook interventions in UP and two other states with a focus on public-private partnership models such as voucher schemes, working with community health workers, mobile health clinics and quality assurance activities [3]. IFPS was designed to complement and support the National Rural Health Mission (NRHM) launch and rollout; the NRHM is a national initiative to address health issues in rural India which was launched in 2005 [3]. Despite the far-reaching contributions of IFPS, the program was not rigorously evaluated, and therefore, conclusions cannot be made regarding its success in influencing increasing FP use over the period. At this juncture, to attain the proposed increase in new users, the GoI and the Government of Uttar Pradesh (GoUP) need to pursue scale-up of proven strategies. The Urban Health Initiative (UHI) that took place between 2009 and 2015 in urban UP provides a wealth of new information on effective implementation strategies that can inform the GoI and GoUP approaches.

This paper presents recent evidence from the rigorous impact evaluation of the UHI. It is divided into the following sections: Section 2 presents the UHI program objectives and key activities. Section 3 presents the evaluation data and methods, and Section 4 presents the results. The last section discusses the results and provides programmatic recommendations for the GoI and other stakeholders seeking to increase mCPR in settings like urban India.

## 2 Urban Health Initiative

The UHI, funded by the Bill & Melinda Gates Foundation (BMGF) and launched in 2009 by a consortium of partners led by FHI360, implemented FP programs in urban areas of UP, India. UHI was designed to complement and support national and state urban health strategies. UHI's goals were to increase mCPR, reduce maternal and infant mortality and use evidence-based strategies aligned with GoI programs to ensure sustainability. UHI objectives were to (a) improve the quality of FP services in high-volume facilities, (b) develop cost-effective interventions to integrate FP with postpartum and postabortion services, (c) test innovative private-sector approaches to increase access to and use of FP by the urban poor, (d) develop interventions to sustain demand for quality contraceptive services and supplies and (e) advocate for funding and a supportive policy environment for FP supplies and services. The main activities of UHI included provision of postpartum and postabortion FP, training providers to improve technical competence and client–provider interactions, expanding the role of the private sector in FP service provision, using community health workers (CHWs) for outreach efforts, and using mid- and mass-media to promote demand for FP services. A key UHI strategy was to task CHWs with visiting every home in slums of target cities to offer information about FP methods; counsel on postpartum FP; accompany women to a health facility; refer women to a health facility or fixed service days; and, if requested, provide short-term methods (pills and condoms).

## 3 Data and methods

The Measurement, Learning & Evaluation (MLE) project, also funded by BMGF, is led by the Carolina Population Center at the University of North Carolina at Chapel Hill. The MLE project was tasked with developing a rigorous evaluation of the UHI; longitudinal data were collected from women and facilities in six cities in UP. Data presented here come from MLE's baseline (2010) and follow-up survey 4 years later in 2014 (termed *endline*) [4]. Between January and August, 2010 representative samples of women in each of six cities (Agra, Aligarh, Allahabad, Gorakhpur, Moradabad and Varanasi) in UP were approached for baseline data collection. A two-stage sampling design was used. In the first stage, random, equal-sized samples of primary sampling units (PSUs) were selected from slum and nonslum areas, permitting oversampling of the poor [5]. All women aged 15–49 and currently married who had spent the previous night in selected households were eligible for baseline interview. At the 2014 endline follow-up, all usual residents interviewed at baseline were eligible for interview. At endline, extensive tracking procedures were used to find women interviewed at baseline in their baseline location or in the location they moved to, assuming it was within one of the six study cities. Overall, we had a final endline reinterview rate of 84%. All study procedures were approved by the Institutional Review Board (IRB) at the University of North Carolina at Chapel Hill, the IRB at the International Center for Research on Women and MAMTA-Health Institute for Mother and Child in India IRB committee.

The dependent variable for this analysis is current modern contraception use. Women who report that they or their husband is using female or male sterilization, an intrauterine device, injection, oral contraceptive pills, female or male condom, lactational amenorrhea, implants, emergency contraception, dermal patch, diaphragm, Standard Days Method or spermicide are considered modern method users. Modern method users are coded 1; traditional method users and nonusers are coded 0.

A number of key program exposure variables are considered in this analysis. All women were asked about exposure to UHI radio and television messages which promoted use of modern FP methods. The UHI program aired three spots on the radio and three similar spots on the television; each woman was asked if she was familiar with each of these spots. At endline, women who reported hearing or seeing any of the spots are coded 1 for the UHI radio or UHI television variables, respectively, and 0 otherwise. All women are coded 0 at baseline since there were no UHI radio or television programs yet.

We also consider mid-media activities such as magic shows, street plays and road shows. A mid-media variable is coded 1 at endline if the woman reported ever being exposed to a mid-media event that discussed FP and 0 otherwise. Women were also asked about exposure to a number of print media including brochures and billboards/posters/wall paintings about FP. Women who reported ever seeing brochures on FP are coded 1 and 0 otherwise (likewise for a billboard/poster/wall paintings exposure variable). As with UHI radio and television, these variables are coded 0 at baseline. The mid-media and print media variables are not specific to UHI in this analysis because the program intentionally integrated activities into those of the government. Because these were not asked at baseline, it is not possible to examine change in these variables between baseline and endline, and thus, we may be overestimating the effect of these variables on modern method use over time.

Another key UHI activity involved outreach in slums by CHWs. Notably, other programs, including government initiatives, use CHW, and it was not possible to distinguish UHI CHW and non-UHI CHW. At baseline and endline, women were asked if they were exposed to a CHW in the last 3 months. We use a community-level CHW exposure variable to capture community presence of CHWs at baseline and endline in the community, here defined as the *PSU/cluster*. This variable is created as a non-self-weighted cluster average of the number of positive responses to exposure to a CHW in the last 3 months divided by the total number of women in the PSU at baseline or endline.^1^ The community-level CHW variable thus represents the percentage of women who reported exposure to a CHW at each panel.

Other general (i.e., non-UHI specific) FP-related variables include whether or not the woman participated in the last 3 months in a community group that discussed FP (measured at endline). Because this was not measured at baseline, all women are coded 0 at baseline; as for the variables above, this variable may overestimate the effects on modern method use. At baseline and endline, women were also asked about exposure to FP on the radio or television in the last 3 months; this is not specific to UHI programming. There are separate general radio and television exposure variables for each time point.

UHI supply variables in the model capture whether women live within a specific distance of a facility where UHI operated. Four variables are included: exposure to a public facility within 1 kilometer (km) with UHI full implementation, exposure to a public facility within 1 km with UHI partial implementation, exposure to a private facility within 1¾ km with UHI full implementation and exposure to a private facility within 1¾ km with UHI partial implementation. The distance band for private facilities accounts for greater willingness to travel for private services.^2^ Full implementation of UHI activities includes strengthening the active referral system; training and mentoring of providers; and enhancing information, education and communication (IEC) materials. Partial implementation includes provision of some training and/or provision of IEC materials.

Multivariable analyses exploit the longitudinal data with fixed-effects regression models with the pooled samples (baseline and endline) to control for both the possible endogeneity of recall to the exposure variables and program targeting of underserved areas. Fixed-effects methods remove time-invariant unobservables for either the respondent or the community that could bias estimates of program impact. Fixed-effect analyses control for the clustered sampling design. The final model specification includes all of the relevant UHI exposure variables measured in the endline survey. Following initial model runs, a small number of variables were removed or regrouped to address high correlation across program activities; the removed variables were not significant in any of the models. All models control for standard control variables related to socioeconomic status that have been used in prior analyses that examine modern method use in the UP context [6] These include age, education, caste, wealth, religion, marital status, ownership of a television or radio, and baseline city of residence. Weighted distributions of these variables can be seen in Table 1.

## 4 Results

Eighty-four percent of women eligible for endline interview were tracked and interviewed (Table 1). As expected, women are older by endline. Column 2 of Table 1 demonstrates that, for most of the demographic characteristics, we were able to interview between about 78%–86% of the eligible women; this suggests that nonresponse and attrition are not biasing the data in any specific direction.

Table 2 presents the percentage of women exposed to the UHI and general FP program activities at baseline and endline. At endline, more than four fifths of women reported ever seeing a billboard/poster/wall painting with an FP message. Sixty-one percent of women recognized UHI-specific messages in television programs. About a quarter of women reported exposure to FP brochures at endline. There was high exposure to general FP messages on the television in the last 3 months at baseline and endline (about 70% in both panels). At baseline, in the average cluster, 65% of women reported exposure to CHW in the last 3 months; by endline, this percentage had declined to 45%. Exposure to the other activities including community groups, UHI radio and mid-media activities was below 5% at endline. Also presented in Table 2 is the percentage of women exposed to UHI full and partial service delivery activities within 1 km of a public or 1¾ km of a private facility. About a quarter of women live within 1 km of a public facility where UHI implemented program activities. A greater percentage of women live within 1¾ km of a private facility where UHI operated.

Table 3 shows contraceptive use at baseline and endline. At baseline, 49% of women were using a modern method and 15% a traditional method. By endline, there is greater modern method use (54%) and traditional method use declined slightly (13%). Female sterilization is dominant in both panels, with the remainder of use being condoms or traditional methods. Also presented in Table 3 are the changes in method use between baseline and endline. Overall, 25% of women changed their behavior; 15% became modern method users, while 10% abandoned modern method use. The remaining women either continued a modern method (38%) or remained nonusers or traditional method users (36%). Given that this is a longitudinal sample and, over time, respondents age and may be more likely to adopt a modern method, multivariate analyses are needed to examine how observed changes over time relate to program exposure.

Table 4 presents the multivariable fixed-effect impact results expressed as marginal effects (MEs). MEs indicate the average expected change in modern method use if each woman in the sample went from unexposed (to the particular program intervention) to exposed. This is a useful way to understand the average potential impact of an activity if all women were exposed to that activity. The ME of exposure to brochures is the largest at 7.0% (ME = 6.96, p < .001). Exposure to FP billboards/posters/wall paintings results in increased modern method use such that if all women went from being unexposed to exposed, we would expect about a 2.1 percentage point increase in modern method use (ME = 2.09, p < .05). Furthermore, greater community-level exposure to CHW leads to greater modern method use (ME = 1.66, p < .10); although this effect was only borderline significant. Supply-side effects are also observed. In particular, if all women lived within 1 km of a UHI full implementation public facility, use would be 2.5 percentage points higher (ME = 2.48, p < .05). We also find a borderline effect for living within 1¾ km of a partial implementation private sector facility (ME = 1.56, p < .10). Finally, if all women had been exposed to any FP messages on the television (whether or not UHI), modern method use would be about 2.5 percentage points higher (ME = 2.46, p < .001).

A number of the program variables were not significant in our models. These included mid-media and community group activities; exposure to UHI radio and UHI television was also insignificant (regardless of whether we controlled for general radio and television exposure). This latter result is not surprising given that the UHI radio and television activities were halted in the second half of the UHI program.

## 5 Discussion

These results demonstrate impacts of the UHI program on modern method use across the six cities in UP where programs were implemented and evaluated. Our results are the first for urban settings in India to show program impacts and relative contribution of different strategies for both demand-side and supply-side activities on modern contraception use. Previous studies from India have shown some positive associations between modern contraceptive use and either demand-side (e.g., interpersonal communication and mass media) or supply-side (e.g., activities to improve the quality of services offered) program activities [7], [8], [9], [10]. Our study, however, is the first to use a rigorous design with longitudinal data at both the individual and facility levels to demonstrate impacts of a multicomponent FP program.

It is worth noting that the program activities that were significant in this study are the types of activities typically included in FP programming in India and elsewhere. What is noteworthy is the comprehensive approach taken by the UHI program to implement these activities with a particular focus on meeting the needs of urban women and specifically urban poor women. In particular, the program used field-generated inputs to develop a full set of communication materials for each type of FP method. The set of materials included handouts such as counseling cards, fact sheets and frequently asked questions (all considered as brochures/print materials) as well as posters and wall paintings that included similar information by method. The materials all had similar branding and consistent messages that were meant to complement the GoI (and GoUP) FP strategies. These communication materials were used by CHWs for counseling in health facilities and appeared as posters on walls in facilities and communities. In addition, the program specifically targeted the urban poor by having CHWs map slum areas and undertake intensive outreach efforts within every slum identified and mapped. Future programs in urban India should consider including the significant activities found in this study (e.g., brochures, billboards/posters/wall paintings, CHW, supply side and television) and use local-level targeting of poor areas to create comprehensive, multicomponent programs; these programs should also ensure that activities are linked, as done by UHI, to support broader exposure and impact.

The findings from this study can be used by the GoI to inform strategies to attain their FP 2020 commitments and to support the new National Urban Health Mission (NUHM) which is a sub-mission under the National Health Mission; the NUHM was launched in 2013 with the goal to provide primary health care services particularly to the urban poor [11]. The activities of the UHI have been developed to fit squarely into the GoI's commitment to provide primary health care, which includes FP, to urban poor populations. This means that the effective strategies of the UHI program including promoting FP use through billboards/posters/wall hangings, brochures, CHWs and the television and improving the supply environment through training and mentoring providers, supporting active referral and provision of IEC materials are likely to be sustainable and scalable to other cities in UP and other states and urban areas in India.

## Figures and Tables

**Table 1:** Characteristics of women surveyed at baseline (2010) and endline (2014) in six urban sites in UP, India

	Baseline^a^ distribution (%)	Percent interviewed at endline (%)	Distribution among endline sample (%)
2010^b^	2014	2014^b^
Characteristic	(*n* = 16,802)		(*n* = 14,026)
Age at baseline			
15–19	2.3	80.7	0.1
20–24	13.6	79.5	4.4
25–29	20.2	81.8	16.0
30–34	19.7	82.6	20.3
35–39	18.8	85.1	19.4
40–44	14.8	86.6	18.1
45–49	10.6	88.2	13.9
50 +	NA	NA	7.8
Education at baseline			
No education	32.9	83.1	31.6
1–5 years education	9.9	85.9	9.9
6–8 years education	11.7	84.6	11.6
9–12 years education	23.7	84.7	24.2
13 + years education	21.8	81.2	22.7
Wealth status at baseline			
Poorest	17.6	79.5	16.1
Poor	19.1	83.2	18.8
Medium	20.2	84.2	20.3
Rich	21.3	86.0	22.5
Richest	21.8	85.7	22.4
Caste			
Scheduled caste	18.1	86.4	18.0
Scheduled tribe	0.3	79.6	0.3
Other backward caste	42.8	84.2	42.6
General caste	38.6	81.1	38.9
No caste	0.1	80.8	0.2
City			
Agra	23.7	80.1	22.8
Aligarh	12.1	85.3	12.5
Allahabad	18.8	85.5	18.9
Gorakhpur	15.4	84.1	15.7
Moradabad	9.5	83.9	9.7
Varanasi	20.5	82.8	20.3
Religion			
Hindu	77.4	83.4	77.5
Muslim	21.6	84.1	21.5
Christian	0.3	76.7	0.3
Sikh	0.4	82.8	0.5
Jain	0.2	83.3	0.3
Ownership of radio or television			
Yes	86.3	84.7	94.1
No	13.7	78.3	5.9
Total	100%	84%	100%

NA, not applicable.

a At baseline, all women interviewed were married. Baseline sample only includes household usual residents; visitors who were not eligible for follow-up are not included.

b Baseline estimates use baseline weights; endline estimates use endline weights.

**Table 2:** Exposure to UHI program and other FP activities at baseline (2010) and endline (2014) among women surveyed at both time periods in six urban sites in UP, India (*n* = 14,026)

	Baseline %	Endline %
Exposure	2010	2014
Percentage of women ever exposed to:		
UHI radio program^⁎^	0.00	2.56
UHI television program^⁎^	0.00	61.40
Mid-media activities (magic shows, caravans, etc.) discussing FP^⁎^	0.00	3.54
Brochures on FP^⁎^	0.00	23.45
Billboards on FP^⁎^	0.00	80.79
Community group activities on FP^⁎^	0.00	0.83
Percentage of women exposed to:		
General FP messages on the radio in last 3 months	7.22	1.44
General FP messages on the television in last 3 months	70.34	69.49
Mean percentage of women in community (PSU) exposed to^a^:		
Community health workers in the last 3 months	65.08	44.46
Percentage of women living in a community (PSU) within:		
1 km of a UHI full implementation^b^ public facility^⁎^	0.00	29.26
1 km of a UHI partial implementation^c^ public facility^⁎^	0.00	23.88
1¾ km of a UHI full implementation^b^ private facility^⁎^	0.00	56.90
1¾ km of a UHI partial implementation^c^ private facility^⁎^	0.00	38.72

^⁎^Not measured or does not exist at baseline; set to 0 at baseline.

a Mean value is non-self-weighted mean.

b Full implementation was defined by the program as being UHI strengthened or supported facilities to do active referral, UHI provided training and mentoring, and UHI provided IEC materials.

c Partial implementation was defined by the program as being facilities that received UHI training and/or IEC materials.

**Table 3:** Contraceptive use at baseline (2010) and endline (2014) among women surveyed at both time periods in six urban sites in UP, India

	Baseline%	Endline%
	2010	2014
Contraceptive use	(*n* = 14,026)	(*n* = 14,026)
Nonuser	36.00	33.20
Modern method user	48.76	53.75
Traditional method user	15.23	13.06
Method mix (among users)	(*n* = 8,977)	(*n* = 9,369)
Sterilization (female or male)	35.72	42.00
IUD	4.34	6.13
Oral contraceptive pill	4.73	5.25
Condom	29.85	25.79
Other modern method^a^	1.45	1.28
Traditional method	23.81	19.55
Transitions in use between baseline and endline	(*n* = 14,026)	(*n* = 14,026)
Nonuser or traditional user both times	NA	36.11
Nonuser or traditional user to modern user	NA	15.13
Modern user to nonuser or traditional	NA	10.14
Modern user both times	NA	38.62

a Other modern methods include lactational amenorrhea, implants, emergency contraception, dermal patch, diaphragm, Standard Days Method, and spermicide.

**Table 4:** Fixed-effects marginal effects (ME) of program activities on modern method use among women surveyed at baseline (2010) and endline (2014) in six cities of UP, India

	MEs of 100% program exposure		
Characteristic	Change in CPR (%)	(SE %)	p value
UHI radio program	0.70	(2.35)	
UHI television program	0.63	(0.86)	
Mid-media activities (magic shows, caravans, etc.) on FP	− 0.99	(2.17)	
Brochures on FP	6.96	(1.13)	***
Billboards on FP	2.09	(0.86)	*
Community group activities on FP	2.52	(4.58)	
Mean exposure to community health workers	1.66	(0.99)	+
Live within			
1 km of a UHI full implementatio^a^ public facility	2.48	(1.03)	*
1 km of a UHI partial implementation^b^ public facility	− 0.73	(1.03)	
1¾ km of a UHI full implementation^a^private facility	− 0.08	(0.92)	
1¾ km of a UHI partial implementation^b^private facility	1.56	(0.90)	+
General FP messages on the radio	− 0.46	(1.63)	
General FP messages on the television	2.46	(0.71)	***

Note: Model controls for age, education, wealth, city, caste, religion, marital status and ownership of a television or radio; MEs if 100% of women exposed.

+ p < .10; *p < .05; **p < .01; ***p < .001.

a Full implementation was defined by the program as being UHI strengthened or supported facilities to do active referral, UHI provided training and mentoring, and UHI provided IEC materials.

b Partial implementation was defined by the program as being facilities that received UHI training and/or IEC materials.

## Footnotes

1 Less than 5% of the women in the endline sample had moved; these women receive the community-level variable for their baseline location at both time points.

2 Sensitivity analyses suggested that a larger distance band was appropriate for private facilities.
